# Long-Term Survival Following Multimodal Therapy with Sunitinib and Surgery for Recurrent Duodenal Gastrointestinal Stromal Tumor

**DOI:** 10.70352/scrj.cr.25-0260

**Published:** 2025-06-27

**Authors:** Manatsu Mizuno, Tsuyoshi Takahashi, Yoshito Tomimaru, Yukinori Kurokawa, Takuro Saito, Takaomi Hagi, Kota Momose, Kotaro Yamashita, Koji Tanaka, Tomoki Makino, Kiyokazu Nakajima, Tomomi Fujii, Eiichi Mori, Hidetoshi Eguchi, Yuichiro Doki

**Affiliations:** 1 Department of Gastroenterological Surgery, The University of Osaka Graduate School of Medicine, Suita, Osaka, Japan; 2 Department of Pathology, The University of Osaka Graduate School of Medicine, Suita, Osaka, Japan

**Keywords:** gastrointestinal stromal tumor (GIST), *KIT* mutation, sunitinib, drug resistance, multimodal treatment, surgical intervention, progression-free survival

## Abstract

**INTRODUCTION:**

Gastrointestinal stromal tumors (GISTs) are mesenchymal tumors often driven by *KIT* or *PDGFRA* mutations. Sunitinib, a second-line tyrosine kinase inhibitor (TKI), is effective in imatinib-resistant cases, particularly those with secondary *KIT* mutations in the ATP-binding domain. However, resistance to sunitinib poses challenges, and evidence supporting surgery during sunitinib treatment remains limited.

**CASE PRESENTATION:**

A 77-year-old male presented with multiple liver and peritoneal metastases from a duodenal GIST. Initial treatment with imatinib was discontinued due to adverse effects, and sunitinib was initiated, maintaining disease stability for 7 years. Disease progression as a solitary lesion in liver segment seven was identified via imaging and diagnosed as sunitinib-resistant. A laparoscopic partial hepatectomy was performed, achieving complete resection without complications. Postoperative resumption of sunitinib has controlled all other lesions for 9 years with no recurrence.

**CONCLUSIONS:**

This case highlights the potential of combining sunitinib with surgery to manage drug-resistant GIST. Localized surgical resection of resistant lesions, integrated with systemic therapy, may offer prolonged survival in selected patients. Further studies are needed to define the optimal role of surgery in this context.

## Abbreviations

FDG-PET
: fluorodeoxyglucose-positron emission tomography
GIST
: gastrointestinal stromal tumor
KIT
: tyrosine-protein kinase KIT (CD117)
OS
: overall survival
PDGFRA
: platelet-derived growth factor receptor alpha
PFS
: progression-free survival
TKI
: tyrosine kinase inhibitor
SUVmax
: maximum standardized uptake value
VEGFR
: vascular endothelial growth factor receptor

## INTRODUCTION

GISTs are the most common gastrointestinal tract mesenchymal tumors, originating from the interstitial cells of Cajal. These tumors are characterized by activating mutations in *KIT* or *PDGFRA*, which serve as targets for molecular therapies.^1,2)^ Complete surgical resection is the standard curative treatment for localized GISTs; however, the management of recurrent or metastatic GISTs primarily relies on systemic therapies. Molecularly targeted therapies, including imatinib, sunitinib, regorafenib, ripretinib, and pimitespib, have significantly improved outcomes by PFS and OS. Despite these advancements, the median OS for patients with recurrent GISTs remains suboptimal, highlighting the need for novel therapeutic strategies.^3–6)^

Sunitinib, a second-line treatment for GISTs following imatinib failure, is a multitargeted TKI that inhibits KIT, PDGFRA, VEGFRs, and other kinases involved in tumor growth and angiogenesis.^7)^ Sunitinib has demonstrated efficacy in patients with imatinib-resistant GISTs, particularly those with secondary *KIT* mutations in the ATP-binding domain. Clinical studies report a median PFS of 27.3 weeks (approximately 6.3 months) for sunitinib-treated patients, highlighting its importance in prolonging disease control. However, resistance to sunitinib often emerges due to additional mutations in *KIT* or alternative pathways, posing significant challenges.^8)^ This situation highlights the need for adjunctive strategies, such as surgery or alternative systemic therapies, to effectively manage drug-resistant GISTs.

The role of surgery in the management of metastatic or recurrent GISTs remains a topic of debate. Although surgery is the cornerstone of treatment for localized GISTs, its application in advanced disease is less clear due to limited evidence. Retrospective studies have suggested potential benefits of surgery in selected cases, such as those with localized progression or resistance to TKIs, particularly when combined with systemic therapies.^8,9)^ However, the optimal timing, patient selection criteria, and outcomes of such multimodal approaches are not well established. Most existing literature on the surgical treatment of GISTs focuses on patients receiving imatinib. Although reports on the role of surgery during second-line sunitinib treatment are rare, they are crucial for understanding the potential of surgery in managing drug-resistant or progressing GISTs. Combining targeted therapy with surgery may offer an integrated approach to prolong survival and control disease progression.

In this context, our team has actively explored the utility of surgery in patients resistant to molecular therapies, reporting favorable outcomes in selected cases. However, evidence remains sparse, particularly regarding the combination of surgery with second-line therapies such as sunitinib. To address this gap, we present a case demonstrating prolonged survival achieved through a multimodal treatment strategy combining sunitinib and surgical intervention. This report underscores the potential of such approaches to optimize outcomes for patients with advanced GISTs.

## CASE PRESENTATION

**Fig. 1:**
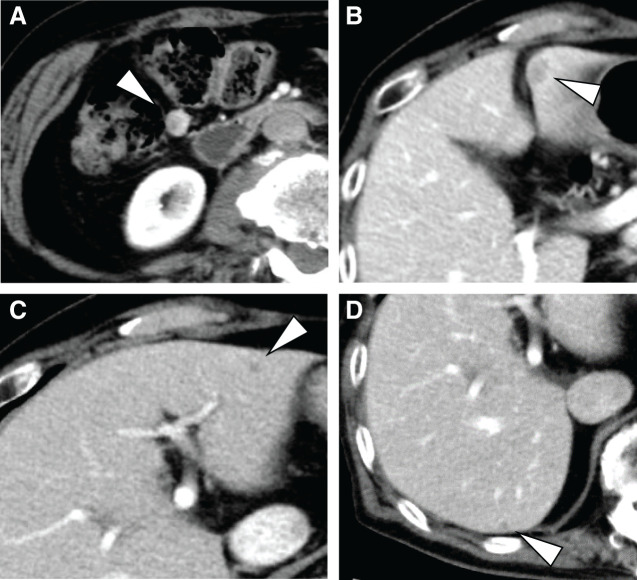
Abdominal CT at initial consultation. (**A**) Disseminated lesion near the duodenum. (**B**) Liver metastasis (S3). (**C**) Liver metastasis (S3). (**D**) Liver metastasis (S7).

**Fig. 2:**
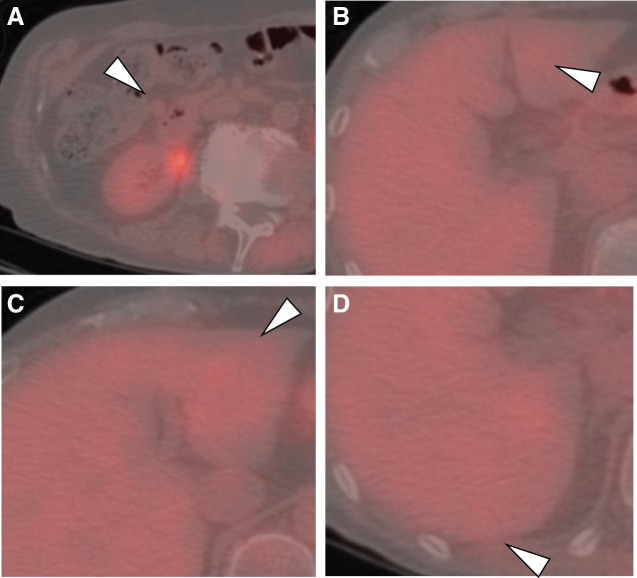
PET-CT image at initial presentation. (**A**) Disseminated lesion near the duodenum. (**B**) Liver metastasis (S3). (**C)** Liver metastasis (S3). (**D**) Liver metastasis (S7). PET, positron emission tomography

A 77-year-old male patient was referred to our hospital after a CT scan revealed multiple liver and peritoneal metastases originating from a duodenal GIST. Four years earlier, the patient had undergone a partial duodenectomy for a primary duodenal GIST. Enhanced CT imaging at the time of referral identified multiple liver metastases and disseminated lesions near the duodenum (**Fig. 1A**–**1D**). FDG-PET/CT performed at the initial consultation showed no significant FDG uptake in either the liver metastases or the peritoneal lesions (**Fig. 2A**–**2D**). Given the presence of multiple lesions, treatment with imatinib was initiated. However, 1 month after starting imatinib, the patient developed a Grade 3 skin rash, a known adverse effect of the drug, necessitating the discontinuation and a subsequent switch to sunitinib.

**Fig. 3:**
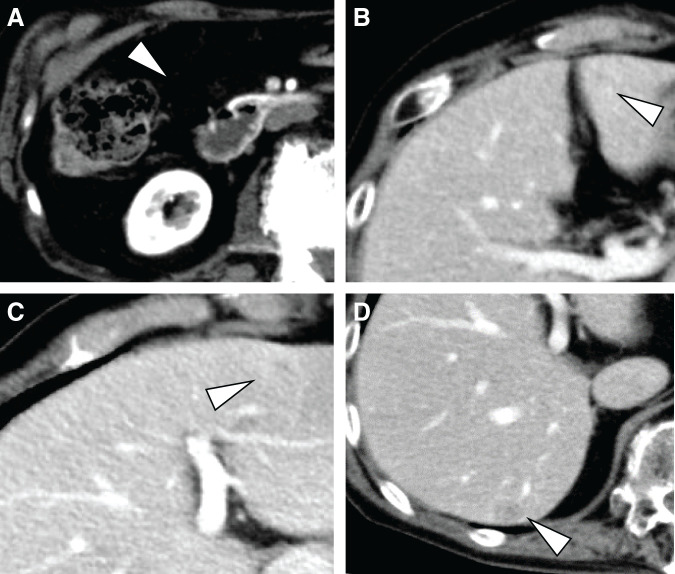
Tumor response to sunitinib and disease progression. (**A**–**C**) Abdominal CT of lesions that responded to sunitinib (arrowhead). (**D**) An abdominal contrast-enhanced CT scan revealed an increase in the size of a lesion in liver segment 7 (S7) (arrowhead).

**Fig. 4:**
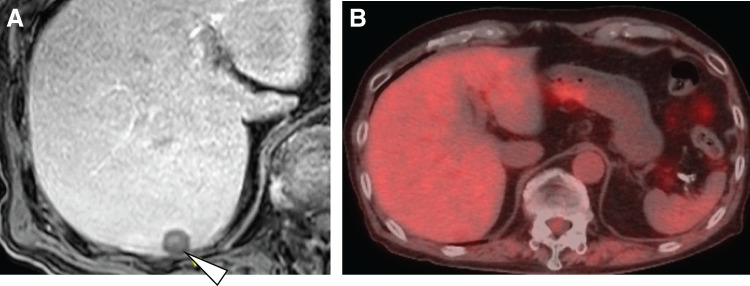
Preoperative imaging of the liver lesion. (**A**) Preoperative MRI imaging. (**B**) Preoperative PET-CT imaging. PET, positron emission tomography

Sunitinib treatment demonstrated effectiveness and maintained disease stability for 7 years. However, at the 7-year mark post-sunitinib initiation, an abdominal contrast-enhanced CT scan revealed that peritoneal dissemination and two other liver metastases remained stable (**Fig. 3A**–**3C**), along with an increase in the size of a lesion located in liver segment seven (S7) (**Fig. 3D**). Similarly, MRI confirmed progressive enlargement of the solitary lesion in liver S7 (**Fig. 4A**), whereas FDG-PET imaging revealed no significant FDG uptake in this region. (**Fig. 4B**). This lesion was diagnosed as a solitary, sunitinib-resistant tumor.

**Fig. 5:**
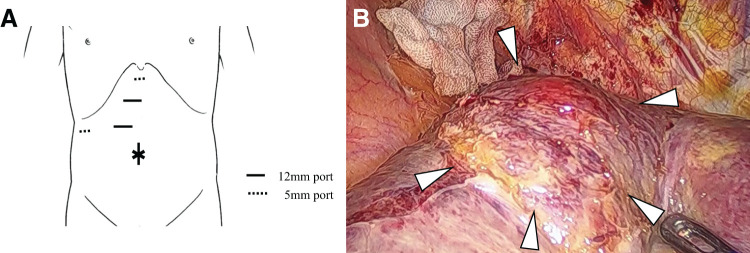
Surgical findings. (**A**) Using the umbilicus as a camera port, the procedure was performed with five ports. (**B**) Tumor in liver segment 7 (S7).

Given the diagnosis of a localized, resistant lesion, the potential benefits of local treatment were considered, leading to the decision to proceed with surgical resection. The patient subsequently underwent laparoscopic partial hepatectomy of S7, with an operative time of 2 h and 49 min (**Fig. 5**). The procedure was uneventful, with minimal blood loss of 30 mL. Postoperatively, the patient experienced no complications and was discharged on the 12th POD.

**Fig. 6:**
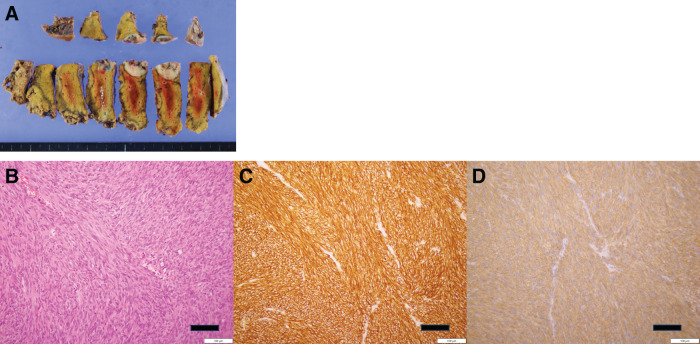
(**A**) The macroscopic findings of the resected liver. (**B**) Pathological findings. Bar represents 100 μm. Hematoxylin and eosin (**B**), Discovered on GIST-1 (**C**) and KIT (**D**) sections of gastric GISTs. GIST, gastrointestinal stromal tumor

Pathological examination of the resected tumor confirmed its identity as a metastatic GIST (**Fig. 6A**), with positive immunohistochemical staining for KIT and DOG1 (**Fig. 6B**–**6D**). Genetic analysis identified a mutation in *KIT* exon 9. Sunitinib therapy was temporarily paused for 2 weeks before and after surgery but was resumed postoperatively.

Nine years of post-sunitinib initiation, all other lesions have remained well-controlled, with no evidence of GIST recurrence.

## DISCUSSION

We report a case of drug-resistant GIST with multiple liver metastases and peritoneal metastases that achieved long-term survival through a multidisciplinary approach combining sunitinib and surgery. In this case, surgical intervention was safely performed without intraoperative or postoperative complications, even during sunitinib administration. Sunitinib treatment has been successfully continuing for over 9 years, including more than 2 years postoperatively.

Regarding imatinib-resistant GIST treatment, surgical resection may be a meaningful option in selected cases. Kikuchi et al. reported that even in cases with multiple secondary mutations leading to resistance against both imatinib and sunitinib, surgical resection should be considered when R0 resection is achievable and disease control with molecular targeted therapy is maintained.^10)^ However, current guidelines don’t recommend surgical intervention strongly due to insufficient evidence supporting its efficacy.^2)^ Most reports on surgical resection for recurrent GISTs involve cases treated with imatinib, and reports during sunitinib therapy are extremely rare. The timing of surgical resection has been discussed through three strategic approaches: (1) primary surgery before initiating drug therapy, (2) surgery during the response phase to drug therapy, and (3) surgery after the development of drug resistance. Primary surgery as an initial treatment has been reported to result in poor prognosis, while evidence demonstrating the utility of the latter two strategies remains insufficient.^11,12)^ DeMatteo et al. reported that patients with metastatic GIST who underwent complete tumor resection after achieving a response with imatinib therapy had a 2-year OS rate of 100%. However, outcomes were more variable in patients who demonstrated localized resistance, and cure rates declined after the emergence of imatinib resistance.^13)^ These findings suggest that while surgical resection may have utility in treating metastatic GIST, further investigation is necessary to determine the optimal timing and patient selection criteria.

In this case, although multiple lesions were observed, only the lesion in liver segment 7 (S7) exhibited drug resistance. While the option of resection of all lesions was considered, the other lesions remained controlled with systemic therapy. To minimize the risk of intraoperative and postoperative complications, a minimally invasive approach was prioritized, and only the lesion showing progression was resected. The interruption of sunitinib treatment was minimized, with a 2-week withdrawal period both before and after surgery. Postoperatively, the patient resumed sunitinib therapy without complications. If further tumor progression occurred, additional surgical resection would have been considered. This case demonstrates that molecularly targeted therapy can be continued safely for more than 2 years postoperatively, underscoring the meaningful role of surgical intervention in patient management.

Raut et al. reported on surgical treatments following sunitinib administration in 50 cases of recurrent GIST. Of these cases, 24 with peritoneal recurrence, 1 with liver metastasis, and 25 with combined peritoneal recurrence and liver metastasis—50% underwent R2 resection. Postoperative complications were observed in 54% of cases, with 8 requiring reoperations and 1 resulting in death.^14)^ These findings indicate the importance of careful patient selection and planning for surgical intervention, particularly during sunitinib therapy, due to its higher risks compared with surgical intervention during imatinib therapy. Sunitinib, a multi-kinase inhibitor targeting VEGFR and PDGFR, can impair vascular function and delay wound healing, raising concerns about postoperative complications.^15)^ In this case, resection of only the progressive lesion ensured a minimal-risk approach. By pausing sunitinib treatment for just 2 weeks pre- and postoperatively, the surgery was performed safely without adverse outcomes, and treatment was resumed promptly.

GIST is classified based on genetic mutations, and its drug responsiveness varies according to genotype. Among GIST cases, mutations in the c-kit gene are the most common, with exon 11 mutations present in 70%–80% of cases.^16)^ *KIT* exon 9 mutations are associated with a higher response rate to sunitinib (58%) and significantly longer progression-free survival compared with exon 11 mutations.^17–19)^ In this case, the presence of a *KIT* exon 9 mutation is consistent with the favorable response to sunitinib. Therefore, when considering the appropriateness of surgical intervention during sunitinib treatment, genetic mutations, such as those in the *KIT* gene, may need to be considered, as they can influence drug responsiveness and overall prognosis.

Kawabata et al. reported a strong positive correlation between SUVmax obtained from FDG-PET/CT images and the mitotic rate of imatinib-resistant lesions, demonstrating that SUVmax could serve as a pathological malignancy marker for imatinib-resistant GISTs.^20)^ Furthermore, among high-risk patients with recurrent GISTs, FDG-SUVmax was significantly elevated, suggesting that PET/CT could act as a potential prognostic marker and be useful in making a surgical decision. In the present case, the absence of FDG uptake and the favorable prognosis align with reports indicating that cases with low SUVmax values have longer OS. This observation further underscores the significance of considering surgical intervention for cases with low SUVmax on PET/CT. These findings highlight that SUVmax evaluation may serve as a critical indicator in understanding the disease state and guiding treatment strategies for imatinib-resistant GISTs.

For cases where long-term survival is expected, surgical intervention, even for sunitinib-resistant GIST, can be a valuable component of the treatment strategy.

## CONCLUSIONS

In conclusion, this case demonstrates that surgical resection, combined with ongoing sunitinib therapy, can be a feasible and effective approach for achieving long-term disease control in patients with drug-resistant GISTs.

## ACKNOWLEDGMENTS

We thank Editage (www.editage.com) for English language editing.

And, we gratefully acknowledge the partial financial support provided by the Osaka Geka Shudankai, which contributed to the publication of this article.

## DECLARATIONS

### Funding

Not applicable.

### Authors’ contributions

MM wrote the initial draft of the manuscript.

TT contributed to the analysis and interpretation of the data and assisted in the preparation of the manuscript.

All other authors contributed to data collection and interpretation and critically reviewed the manuscript.

All authors approved the final version of the manuscript and agreed to be accountable for all aspects of the work, ensuring that questions related to the accuracy or integrity of any part of the work are appropriately investigated and resolved.

### Availability of data and materials

The data that support the findings of this study are available on request from the corresponding author, Tsuyoshi Takahashi. The data are not publicly available because they contain information that can compromise the privacy of research participants.

### Ethics approval and consent to participate

The Human Ethics Review Committee of The University of Osaka Graduate School of Medicine approved the protocol for this retrospective study, and each participant provided signed consent. All procedures were performed following the Declaration of Helsinki.

### Consent for publication

Consent for publication was obtained from all individuals whose data appear in the paper.

### Competing interests

There are no conflicts of interest to declare.

## References

[1] Hirota S Isozaki K Moriyama Y Gain-of-function mutations of c-kit in human gastrointestinal stromal tumors. Science 1998; 279: 577–80. 9438854 10.1126/science.279.5350.577

[2] Corless CL McGreevey L Haley A KIT mutations are common in incidental gastrointestinal stromal tumors one centimeter or less in size. Am J Pathol 2002; 160: 1567–72. 12000708 10.1016/S0002-9440(10)61103-0 PMC1850861

[3] The Japanese Society of Cancer Therapy. the Japanese Gastric Cancer Association, the GIST Study Group / Editors: GIST Clinical Practice Guidelines. 4th ed. Kanehara Shuppan; 2022.

[4] Demetri GD von Mehren M Blanke CD Efficacy and safety of imatinib mesylate in advanced gastrointestinal stromal tumors. N Engl J Med 2002; 347: 472–80. 12181401 10.1056/NEJMoa020461

[5] Kanda T Ishikawa T Hirota S Prospective observational study of imatinib therapy in Japanese patients with advanced gastrointestinal stromal tumors: longterm follow-up and second malignancy. Jpn J Clin Oncol 2012; 42: 578–85. 22523393 10.1093/jjco/hys056 PMC7299430

[6] Yeh CN Wang SY Tsai CY Surgical management of patients with progressing metastatic gastrointestinal stromal tumors receiving sunitinib treatment: A prospective cohort study. Int J Surg 2017; 39: 30–6. 28110026 10.1016/j.ijsu.2017.01.045

[7] Ferrara N Gerber HP LeCouter J. The biology of VEGF and its receptors. Nat Med 2003; 9: 669–76. 12778165 10.1038/nm0603-669

[8] Kelly CM Gutierrez Sainz L Chi P. The management of metastatic GIST: Current standard and investigational therapeutics. J Hematol Oncol 2021; 14: 2. 33402214 10.1186/s13045-020-01026-6 PMC7786896

[9] Kawagoe T Kouzu K Tsujimoto H Surgical resection of imatinib-resistant unresectable recurrent lesion of gastric gastrointestinal stromal tumor after sunitinib therapy – A case report. J Natl Def Med Coll 2020; 45: 100–5. (in Japanese)

[10] Kikuchi H Setoguchi T Miyazaki S Surgical intervention for imatinib and sunitinib-resistant gastrointestinal stromal tumors. Int J Clin Oncol 2011; 16: 741–5. 21394667 10.1007/s10147-011-0208-4

[11] Xia L Zhang MM Ji L Resection combined with imatinib therapy for liver metastases of gastrointestinal stromal tumors. Surg Today 2010; 40: 936–42. 20872196 10.1007/s00595-009-4171-x

[12] Du CY Zhou Y Song C Is there a role of surgery in patients with recurrent or metastatic gastrointestinal stromal tumours responding to imatinib: A prospective randomised trial in China. Eur J Cancer 2014; 50: 1772–8. 24768330 10.1016/j.ejca.2014.03.280

[13] DeMatteo RP Lewis JJ Leung D Two hundred gastrointestinal stromal tumors: Recurrence patterns and prognostic factors for survival. Ann Surg 2000; 231: 51–8. 10636102 10.1097/00000658-200001000-00008 PMC1420965

[14] Raut CP Wang Q Manola J Cytoreductive surgery in patients with metastatic gastrointestinal stromal tumor treated with sunitinib malate. Ann Surg Oncol 2010; 17: 407–15. 19898902 10.1245/s10434-009-0784-y

[15] Mendel DB Laird AD Xin X In vivo antitumor activity of SU11248, a novel tyrosine kinase inhibitor targeting vascular endothelial growth factor and platelet-derived growth factor receptors: determination of a pharmacokinetic/pharmacodynamic relationship. Clin Cancer Res 2003; 9: 327–37. 12538485

[16] Martín J Poveda A Llombart-Bosch A Deletions affecting codons 557–558 of the c-KIT gene indicate a poor prognosis in patients with completely resected gastrointestinal stromal tumors: a study by the Spanish Group for Sarcoma Research (GEIS). J Clin Oncol 2005; 23: 6190–8. 16135486 10.1200/JCO.2005.19.554

[17] Heinrich MC Maki RG Corless CL Primary and secondary kinase genotypes correlate with the biological and clinical activity of sunitinib in imatinib-resistant gastrointestinal stromal tumor. J Clin Oncol 2008; 26: 5352–9. 18955458 10.1200/JCO.2007.15.7461 PMC2651076

[18] Heinrich MC Corless CL Demetri GD Kinase mutations and imatinib response in patients with metastatic gastrointestinal stromal tumor. J Clin Oncol 2003; 21: 4342–9. 14645423 10.1200/JCO.2003.04.190

[19] Debiec-Rychter M Dumez H Judson I Use of c-KIT/PDGFRA mutational analysis to predict the clinical response to imatinib in patients with advanced gastrointestinal stromal tumours entered on phase I and II studies of the EORTC Soft Tissue and Bone Sarcoma Group. Eur J Cancer 2004; 40: 689–95. 15010069 10.1016/j.ejca.2003.11.025

[20] Kawabata K Takahashi T Nishida T ^18^F-fluorodeoxyglucose positron emission tomography-computed tomography as a prognostic marker of imatinib-resistant gastrointestinal stromal tumors. Surg Today 2025 Mar 28. Epub ahead of print. 10.1007/s00595-025-03029-7 40148693

